# Exploring Incision Options for Mandibular Fractures: A Review of Vestibular and Crevicular Approach

**DOI:** 10.7759/cureus.66735

**Published:** 2024-08-12

**Authors:** Archita Chittoria, Raj Kumar, Yogendra Malviya, Aparajita Adurti, Aabhash A Agarwal

**Affiliations:** 1 Oral and Maxillofacial Surgery, Jaipur Dental College, Jaipur, IND; 2 Oral and Maxillofacial Surgery, School of Dental Sciences, Sharda University, Noida, IND; 3 Pedodontics, Jaipur Dental College, Jaipur, IND

**Keywords:** trauma injuries, crevicular incision, mandibular fracture, maxillofacial injury, surgical incision

## Abstract

The approach to mandibular fractures remains a contentious topic, with the vestibular and crevicular approaches being prominent incision options, each offering distinct advantages. This review explores the comparative benefits of crevicular incisions over the vestibular approach, focusing on ease of surgical access, better fracture reduction, and enhanced soft tissue healing. Along with a thorough literature review, a case report is also presented to exemplify the successful implementation of crevicular incision in treating a mandibular fracture, emphasizing on its key advantages, which include decreased risk of nerve injury and enhanced patient comfort, underscoring the clinical benefits of this approach. By synthesizing existing literature and case report findings, this review supports the adoption of crevicular incision as a preferred approach for various mandibular fracture cases. This approach offers clinicians a reduced surgical time, good soft tissue adaptation, improved surgical outcomes, and patient well-being.

## Introduction

The mandible is a common site for both intentional and unintentional trauma because of its prominent position on the face [1]. Mandibular fractures can occur from a variety of incidents, including falls from heights, assaults, auto accidents, sports injuries, and many more [2]. The most frequent cause of mandibular fractures is considered to be traffic accidents. Mandibular fractures can be managed via open or closed methods. The approach that an oral and maxillofacial surgeon prefers to take to a fracture site is determined by various factors, namely, surgical expertise, the patient's aesthetic demands, ease of procedure, and, above all, accessibility [3]. There are two approaches to treating mandibular fractures. One is the extraoral approach, which is carried out with a skin incision that is hidden in a shadow or crease beneath the submandibular or submental region [4]. By maintaining plates or wires away from the contaminated oral cavity, this method preserves a sterile environment. However, some patients may have noticeable scarring after the procedure, and there is also a chance of marginal mandibular nerve injury. The intraoral approach is the usual approach opted for by many surgeons for the majority of mandibular fracture cases.

Using a conventional vestibular incision is the most recommended method for intraoral procedures [5]. However, postoperative complications from this incision are often associated with pain, swelling, trismus [6], wound dehiscence, obliteration of vestibular depth, and, most importantly, damage to surrounding vital structures. The use of a crevicular incision significantly minimizes complications commonly encountered with a vestibular incision. The crevicular incision runs along the gingival crevice, which gives a scalloped appearance. This incision can be used to expose fractures of the body, angle, mandibular symphysis, and maxillary fractures. By using this method, the fracture line's upper and lower limits can be seen due to its wider exposure. Additionally, there is a significant decrease in the risk of bleeding, nerve damage, and scarring.

## Case presentation

**Figure 1:**
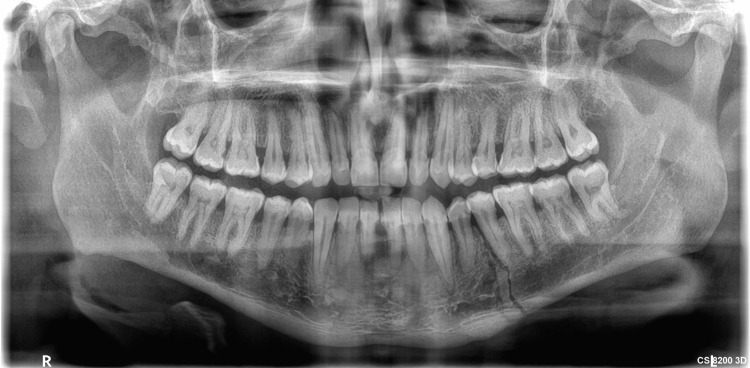
A preoperative orthopantomogram showing an oblique fracture line between teeth 34 and 35, confirming left mandibular body fracture.

A 40-year-old man in overall good health reported to the Department of Oral and Maxillofacial Surgery at Jaipur Dental College, Jaipur, with a history of falling from a bike on the left side of his face eight days prior. He was experiencing pain in the lower left posterior tooth region, difficulty in opening his mouth, and inability to chew food. The clinical examination showed limited mouth opening and mobility in the region between the lower left first and second premolar. An intraoral examination revealed no step deformity in the left body region. Occlusion was undisturbed bilaterally. An orthopantomogram was advised, which showed an oblique fracture line between teeth 34 and 35, confirming the left mandibular body fracture (Figure 1).

**Figure 2:**
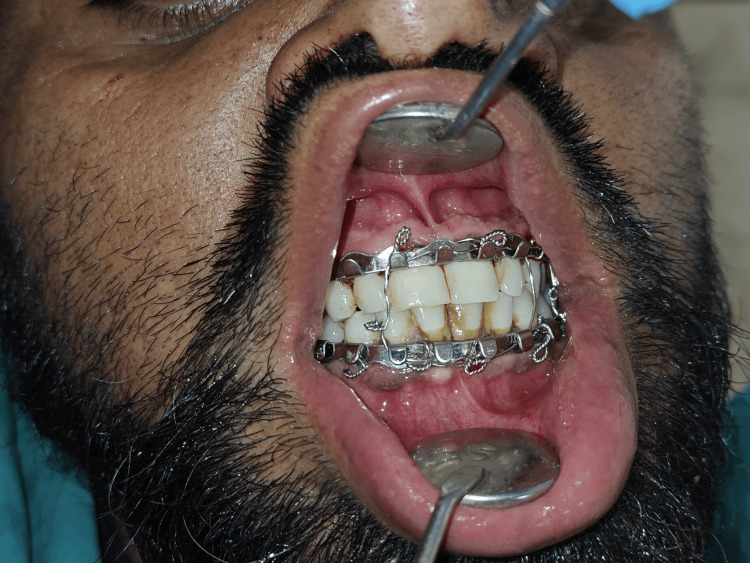
Placement of upper and lower arch bars.

**Figure 3:**
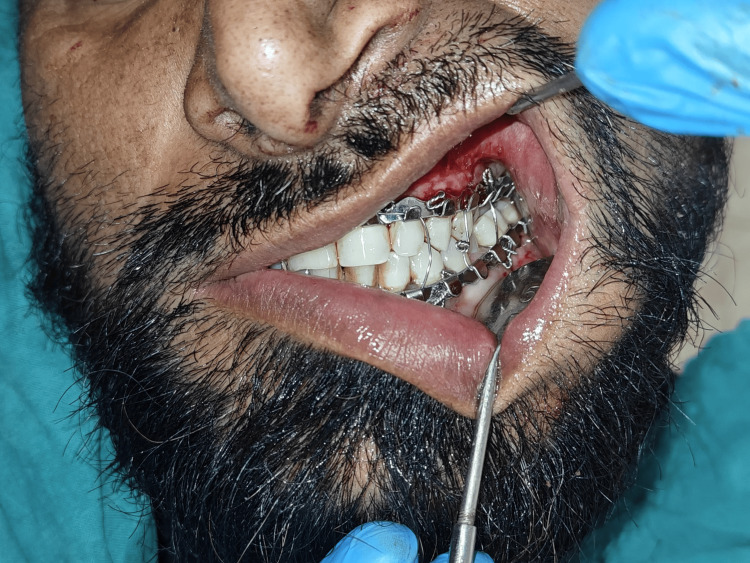
Placement of upper and lower arch bars.

Upper and lower arch bars were placed (Figure 2 and Figure 3), with intermaxillary fixation under local anesthesia. Open reduction and internal fixation under general anesthesia was decided as the treatment plan.

**Figure 4:**
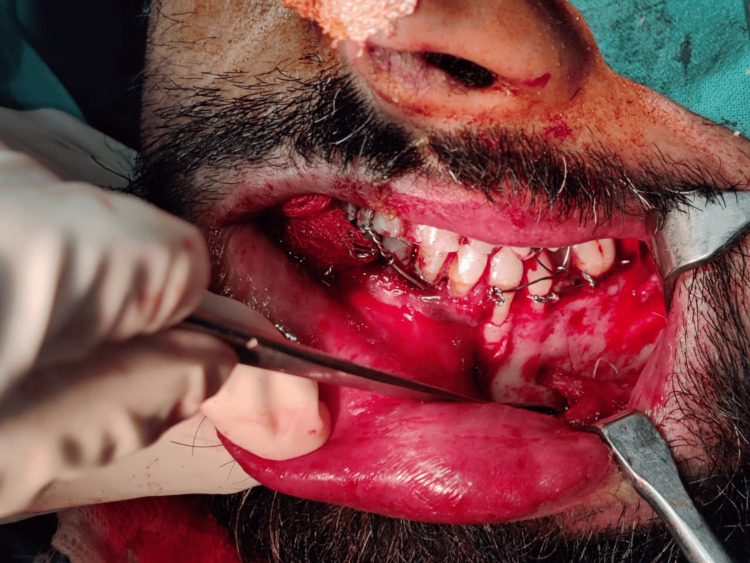
Visualization and isolation of mental nerve, following crevicular incision placement and full-thickness flap reflection.

After induction, a crevicular incision was made extending from the left canine to the left first molar, with an anterior releasing incision given mesial to the canine, exposing the fracture site. A full-thickness mucoperiosteal flap was reflected, following which the mental nerve was carefully identified, dissected, and isolated (Figure 4).

**Figure 5:**
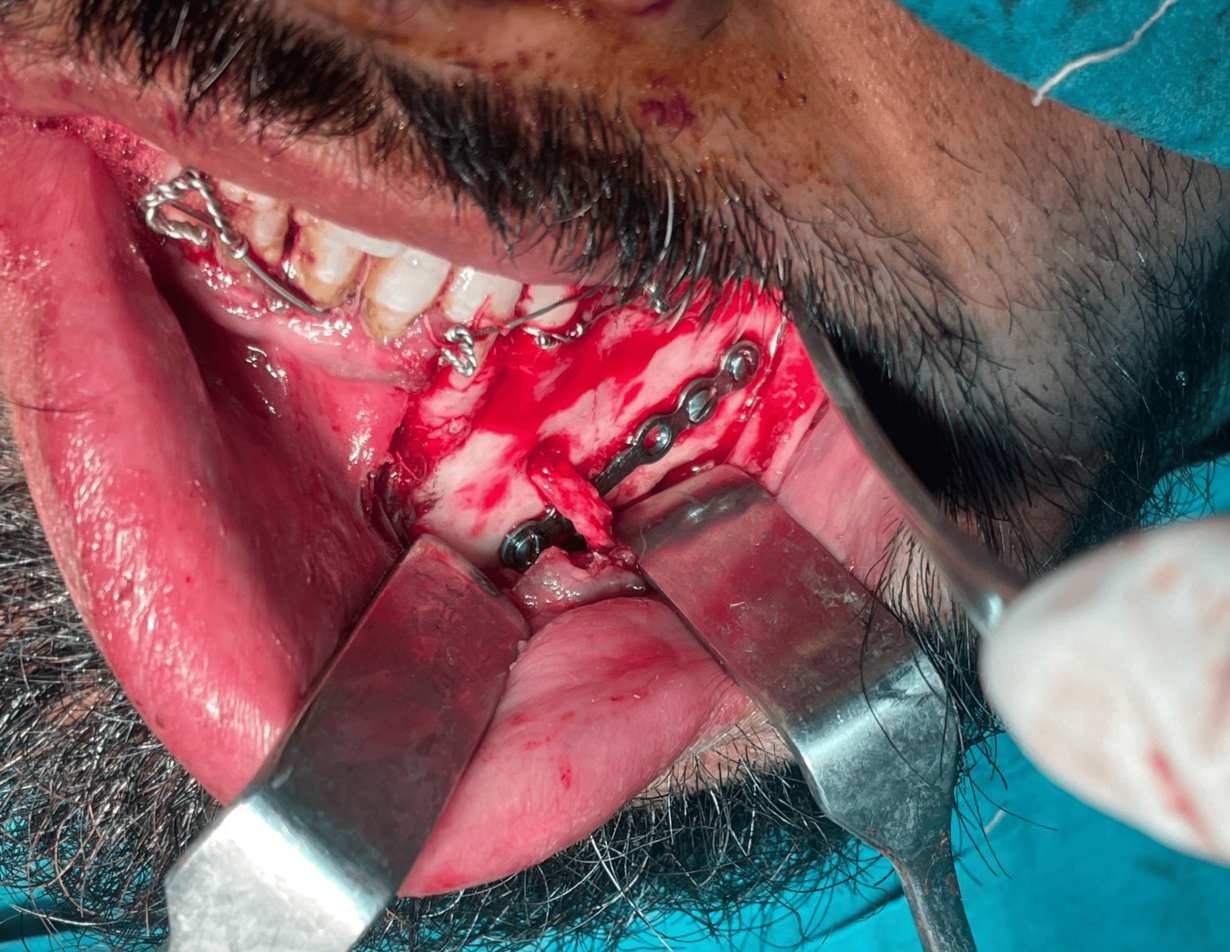
Intraoral access: reduction and fixation with a 2-mm, five-hole-with-gap miniplate and 6-mm screws.

**Figure 6:**
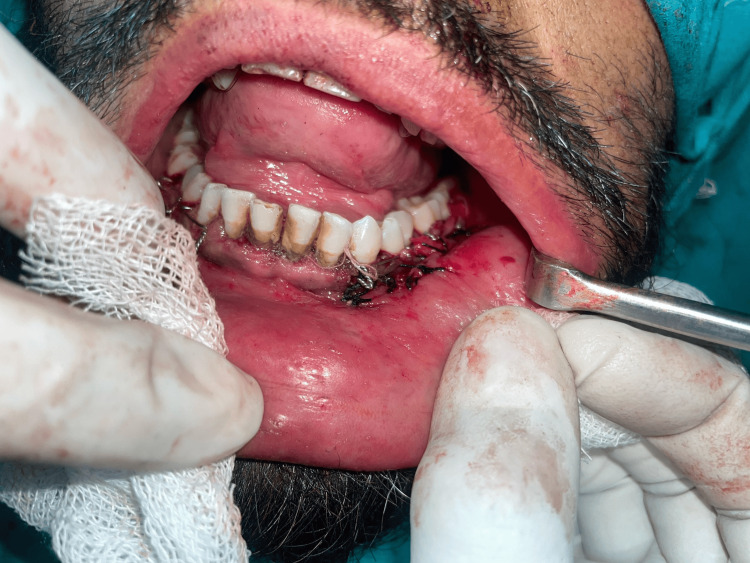
Closure done with 3-0 vicryl sutures.

Good access and visualization of the upper and lower limits of the fracture line were observed. The fractured fragments were reduced into their anatomic position and fixed using a 2-mm, five-hole-with-gap miniplate and 6-mm screws (Figure 5). The incision was closed using 3-0 vicryl sutures (Figure 6).

**Figure 7:**
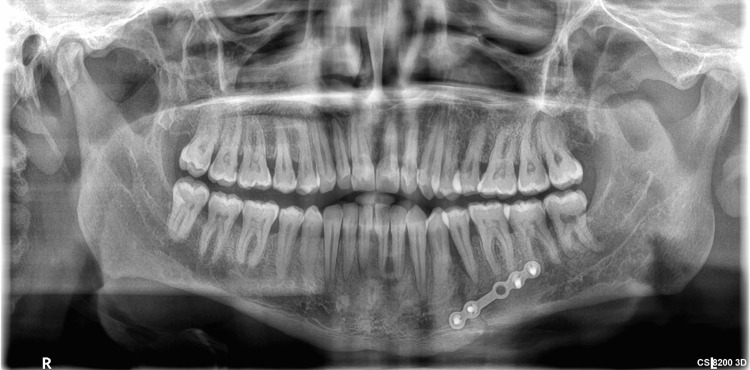
A postoperative orthopantomogram after four weeks, showing adequate bone healing.

**Figure 8:**
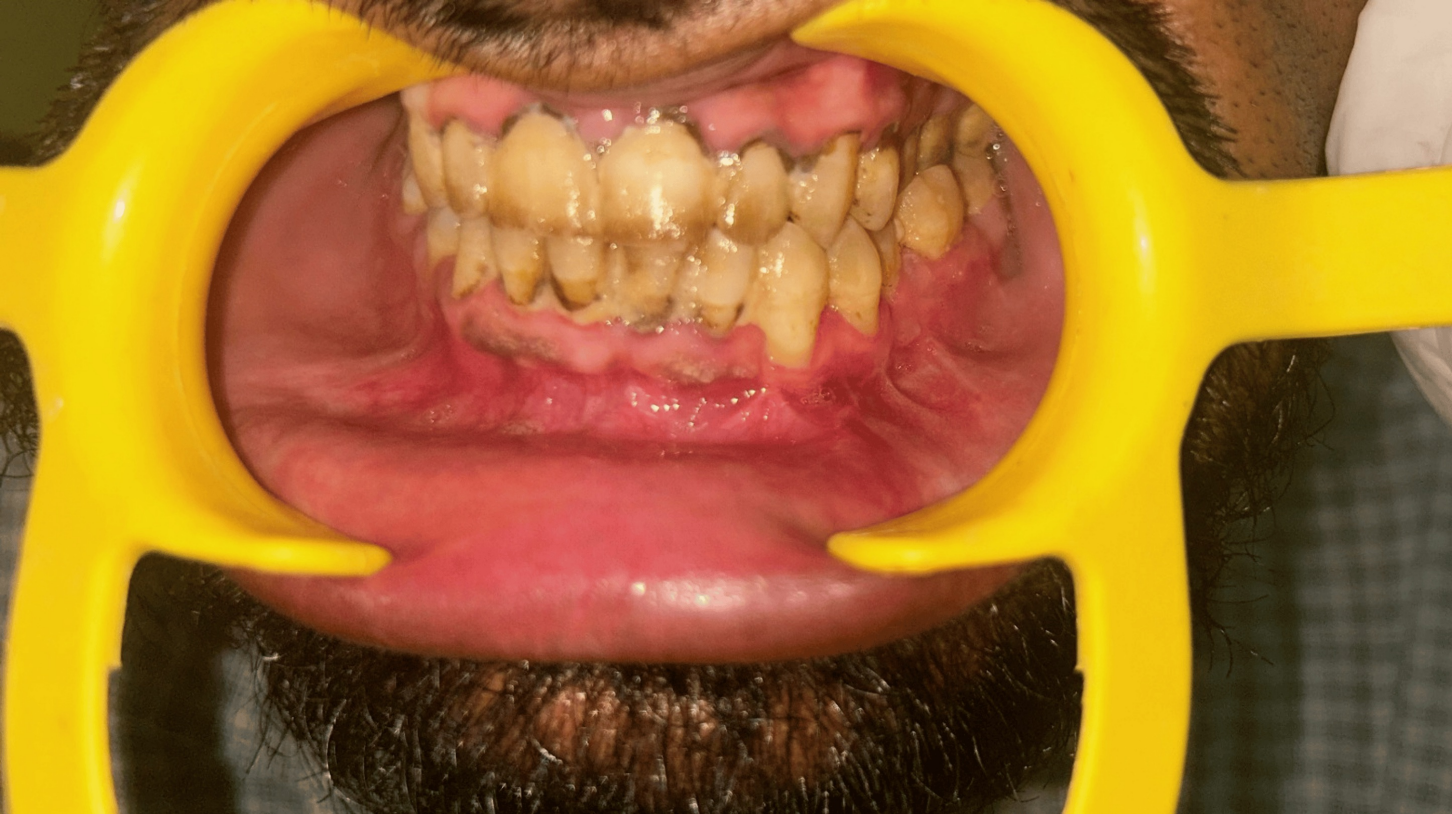
Clinical view of satisfactory soft tissue healing after one month.

An orthopantomogram was done on postoperative day 1, which revealed an adequate reduction of the fractured segments and the correct position of the hardware. The patient was discharged on postoperative day 2. After four weeks, a panoramic view confirmed satisfactory bone healing (Figure 7), and the soft tissue had healed properly as well (Figure 8).

## Discussion

**Figure 9:**
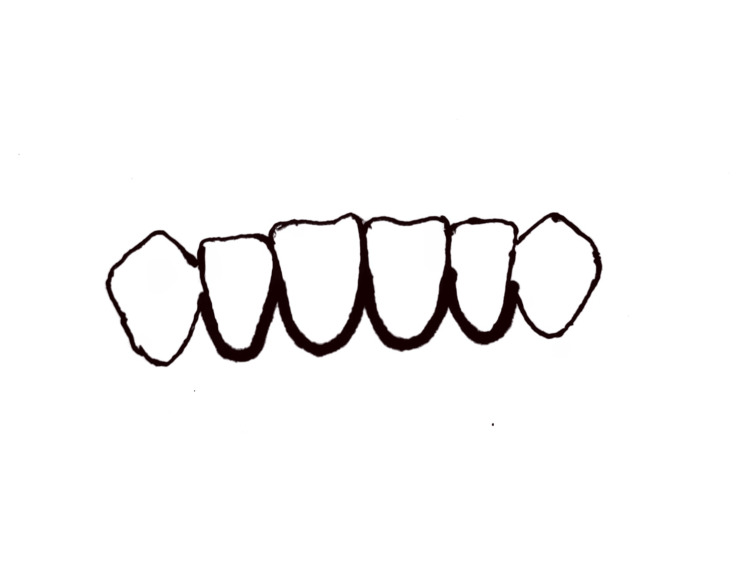
Crevicular incision: incision along the gingival crevices.

The intraoral/transoral approach is a frequently used method for treating mandibular fractures. It involves performing the surgery through a gingiva/oral mucosa incision [3]. Crevicular and vestibular incisions are the two types of intraoral incisions used by surgeons. The gingival crevice is a portion of the scalloped crevicular incision [7], which extends from the base of the pocket to the crest of the bone (Figure 9). It may be given with or without an anterior releasing incision, depending on the exposure required. In the case presented for a body fracture, we used a releasing incision mesial to the canine [4]. 

**Figure 10:**
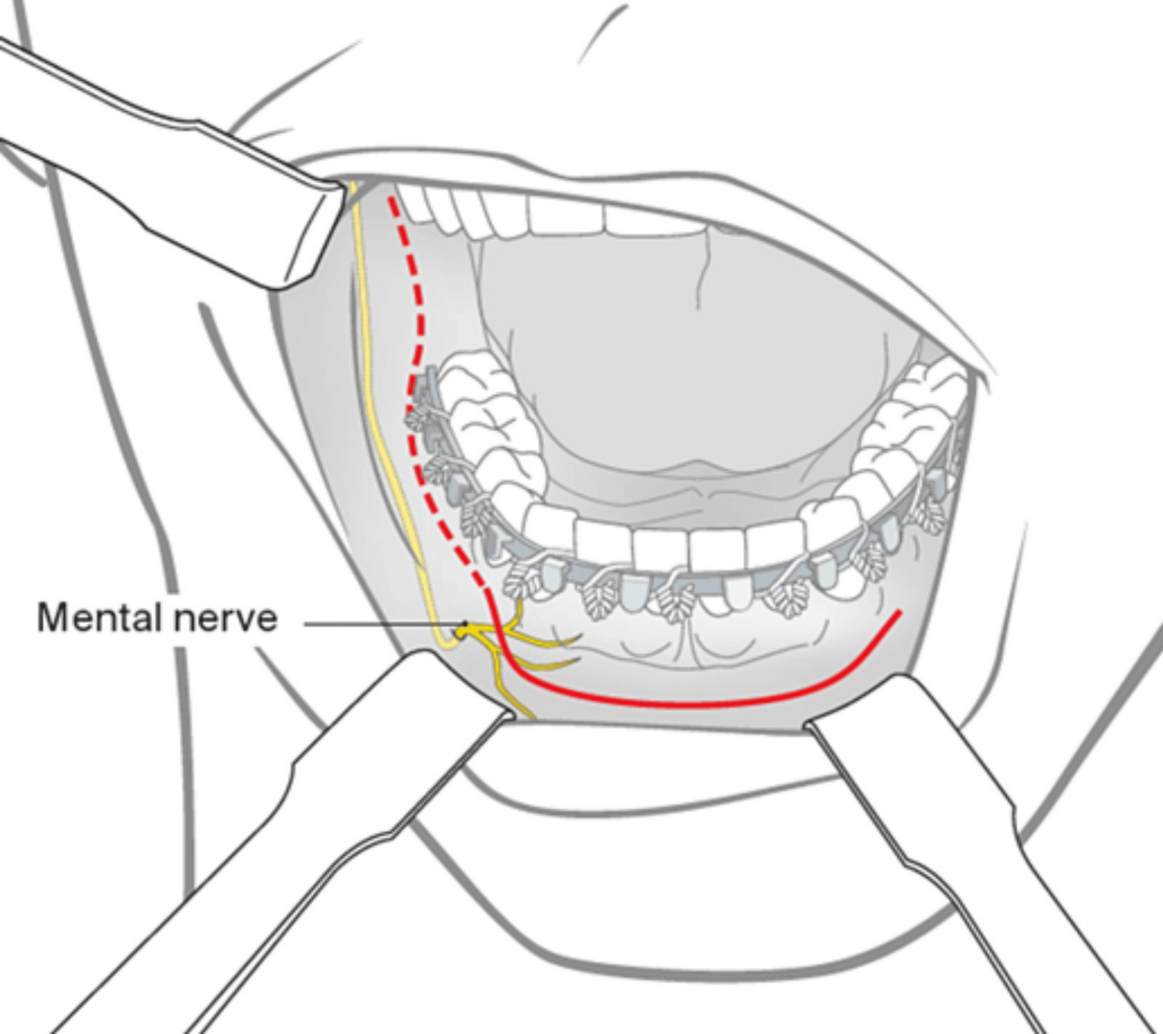
Conventional vestibular incision. Reference: [8]. Permission was obtained from the original source.

Conversely, as shown in Figure 10, the vestibular incision line typically occurs in the mobile gingiva, 8-10 mm from the point where the attached and mobile mucosa converge [8]. One important consideration in intraoral incisions (either vestibular or crevicular) is the presence of mental nerves and vessels exiting from the mental foramen. Utmost care must be taken to protect the nerve from injury during surgery.

Pal et al. [9] proposed a curvilinear modification of the vestibular incision close to the premolar region to protect the mental nerve that exits through the mental foramen from potential injury. However, neurosensory impairment resulting from the conventional vestibular incision remained. The mental nerve, the terminal branch of the inferior alveolar nerve, exits the mandibular bone through the mental foramen below and adjacent to the second premolar. Various researchers have found many variations in the course of the mental nerve, so a good understanding of its branching and distribution is needed for making incisions and dissecting the mental nerve in this region. According to Gray and Clemente [10], the lower lip is supplied by two branches, while the skin above the chin is supplied by one. Kamijo [11] concluded in a study that the mental nerve divides into two branches, the inferior labial and the mental nerve, and as it approaches the angular region, the inferior labial branch divides into angular branches. Hu et al. [12] identified the angular, medial inferior labial, lateral inferior labial, and mental branches as the four branches of the mental nerve.

The position of the mental foramen also plays a vital role while placing an incision, as it is known to have significant anatomical variations. According to Montagu [13], the mental foramen is positioned vertically midway between the base of the mandible and the alveolar process. Matsuda [14] measured the distance between the alveolar process and the superior border of the foramen to be 10.5-18.0 mm. The distance between the inferior border of the foramen and the inferior border of the mandible was found to be 11.5-16.0 mm. The average distance measured by him was 11.89 mm, which is comparable to the reports of the study by von Arx et al. [15] (12.6 mm). The processes of alveolar bone resorption also impact this distance. The foramen is typically found between the premolar apices or by the apex of the second mandibular premolar. Kekere-Ekun [16] described that, atypically, the canine or the first molar can be used to locate the foramen anteriorly or posteriorly. Minor differences in the position of the foramen could also be influenced by race. In the Chinese population, the mental foramen is typically located apical to the second premolar, while in the Caucasian population, it is typically located between the premolars [17,18].

The vestibular incision is given close to the mental nerve, which makes the flap reflection quite difficult. In order to reach the line of osteosynthesis above the mental foramen in the midbody region, one must reflect from below and cross it. Balasubramanian et al. [19] reported that the crevicular incision is superior to the vestibular incision. They suggested that the crevicular incision has fewer postoperative complications, ease of access, better fracture manipulation, minimal surgical time, good wound closure, ease of incision placement even by an inexperienced surgeon, and decreased neurosensory impairment. During a vestibular incision, blind dissection is used to locate and release the nerve from traction. It can be very difficult to visualize the superior and inferior limits of the fracture line, particularly in oblique fractures. This, in turn, makes manipulation and reduction of fractured segments a time-consuming step. In a vestibular incision, blunt dissection parallel to the branches of the mental nerve extends the surgical time. The accumulation of food and debris in the area of the vestibular incision may cause wound infection. As the incision passes through mobile mucosa, it can cause postoperative edema and hinder flap approximation to the bone. Suturing through non-keratinized vestibular mucosa may lead to wound dehiscence.

In contrast, a crevicular incision provides a more direct approach to the bone in a sub-periosteal plane, beginning at the gingival sulcus. Hence, the flap reflection is relatively easy and requires less time. Balasubramanian et al. [19] also emphasized that mental nerve isolation is done in very little time, as the visualization is excellent with this approach. Fractured segments are easily reduced to their anatomic position. Since the miniplate fixation, according to AO principles, is done well away from the line of incision, there is a reduced chance of accumulation of food debris when using a crevicular incision [19]. This subsequently helps in lowering the risk of wound infection or dehiscence. The postoperative triad (pain, swelling, trismus) significantly influences the patient's quality of life. Siddiqui et al. [7] suggested that a crevicular incision, when given with an anterior vertical releasing incision, avoids the accumulation of edematous fluid. This study described that both crevicular and vestibular incisions do not influence periodontal health. Slight gingival recession may be seen with the crevicular incision, which is insignificant. Additionally, the vertical release incision aids in draining inflammatory edema or hematoma, thereby minimizing swelling after surgery. The crevicular incision is a very convenient approach for mandibular fractures, flap surgery, apicoectomy, transalveolar extraction, and bone biopsy procedures [7].

Gapski et al. [20] stated that when gingival protection is crucial, in the case of a shallow vestibule and tense mentalis posture, the crevicular incision is a far better method. Balasubramanian et al. [19] conducted an objective assessment of the gingival position and periodontal status of the involved teeth using Miller's scale when the crevicular incision was used. Till the 14th day following surgery, no statistically significant difference in the gingival position between the pre-surgical and post-surgical stages was found, as concluded by the study.

We have used crevicular incision in various cases of mandibular symphysis, parasymphysis, and body fractures. Intraoperatively, we encountered ease of surgery, better reduction of the fracture, and fixation of hardware compared to the vestibular approach, which was also reported by Balasubramanian et al. [19] in their study. The time taken by us during mental nerve skeletonization was also much less after placing the crevicular incision. Our mean time for the crevicular incision was 11-13 minutes, compared to the vestibular incision, which took an average of 20-22 minutes. There were no instances of nerve transection. Even for less experienced surgeons, the risk of incision-related nerve injury is significantly lower when using a crevicular incision compared to a vestibular approach. Postoperative swelling was quite insignificant when we employed a crevicular incision with a vertical releasing incision, an observation consistent with the investigation carried out by Siddiqui et al. [7]. On the patient's follow-up, no signs of wound dehiscence, such as gaps between the wound margins, pus discharge, pain, or infection of the wound, were observed, which proved the crevicular incision to be superior to the vestibular approach. We checked the surrounding soft tissues and incision line postoperatively after seven days and four weeks, which showed satisfactory results in terms of good soft tissue adaptation and approximation of the incision line. The gingival position was also recorded and evaluated after one month, confirming excellent healing. No periodontal complications were observed in treating mandibular fractures with the crevicular incision. Along with soft tissue, hard tissue healing was also assessed after one month and six months, showing a good amount of bone formation by the end of one month. We have used the crevicular incision in a few malunited fracture cases as well. Ease of access to the inferior border and osteotomy was attained in those cases.

The only issues to be dealt with in the crevicular incision are flap reflection and wound closure when arch bars are fixed. However, this concern can be effectively managed by using eyelets as an alternative if occlusal disturbance is not present.

## Conclusions

The crevicular incision is very simple and easy to use for the operator. It is a very convenient approach for mandibular fractures, flap surgery, apicoectomy, transalveolar extraction, and bone biopsy procedures. It provides proper visualization of the surgical site, less risk of nerve injury, and ease of hardware placement and enables good wound healing compared to the vestibular incision. Hence, we can say that the crevicular incision can be used as a reliable alternative to the conventional vestibular incision in reducing patient discomfort and enhancing good surgical outcomes.
